# Liquid Biopsy in Non-Metastatic Prostate Cancer: Clinical Evidence and Future Directions

**DOI:** 10.3390/cancers18050800

**Published:** 2026-02-28

**Authors:** Maria Chiara Sighinolfi, Giuseppe Pallotta, Marzia Del Re, Koosha Moosavi, Or Schubert, Francesco Rossi, Filippo Gavi, Simone Assumma, Enrico Panio, Angelo Totaro, Filippo Turri, Mauro Ragonese, Nazario Foschi, Pierluigi Russo, Ela Patel, Carlo Gandi, Giuseppe Palermo, Eros Scarciglia, Francesco Pinto, Simona Presutti, Marcio Covas Moschovas, Angelo Minucci, Roberto Iacovelli, Chiara Ciccarese, Luca Tagliaferri, Francesco Pierconti, Camilla Nero, Gian Franco Zannoni, Bernardo Rocco

**Affiliations:** Fondazione Policlinico Agostino Gemelli IRCCS, 00168 Rome, Italy; giuseppe.pallotta17@gmail.com (G.P.); moosavi.kooshamd@gmail.com (K.M.); orshu1993@gmail.com (O.S.); francescorossi1010@gmail.com (F.R.); filippogavimd@gmail.com (F.G.); simone.assumma@gmail.com (S.A.); enrico.panio@gmail.com (E.P.); dr.atotaro@gmail.com (A.T.); giuseppe.palermo@policlinicogemelli.it (G.P.);

**Keywords:** non-metastatic prostate cancer, liquid biopsy, circulating tumor cell, circulating tumor DNA, next-generation sequencing

## Abstract

Background and Objective: Liquid biopsy has transformed the management of advanced prostate cancer, yet its clinical role in non-metastatic disease remains uncertain. Conventional biomarkers such as PSA, imaging, and pathology have limited ability to capture minimal residual disease and biological aggressiveness. The objective of this review was to critically evaluate the current evidence on circulating tumor cells (CTCs) and circulating tumor DNA (ctDNA) in non-metastatic prostate cancer, focusing on feasibility, prognostic value, and potential clinical applications. Methods: A narrative review of PubMed-indexed original studies evaluating liquid biopsy in clinically localized or non-metastatic prostate cancer was performed. Eligible studies included patients treated with curative-intent local therapy or experiencing biochemical recurrence without radiologic metastases. Study designs were predominantly prospective or retrospective observational cohorts. Liquid biopsy analytes included CTCs and ctDNA assessed from peripheral blood plasma using EpCAM-based enrichment, targeted next-generation sequencing, whole-genome sequencing, or ultra-sensitive tumor-informed assays. Primary outcomes included detection rates, associations with clinicopathologic features, biochemical recurrence, metastasis-free survival, and overall survival. Key Findings and Limitations: Across 11 studies, CTC detection using EpCAM-based platforms was infrequent in localized disease and biochemical recurrence and showed limited prognostic value (10–11% in preoperative settings). In contrast, ctDNA was detectable in a minority of patients but consistently identified biologically aggressive disease and a higher risk of recurrence when present, particularly using tumor-informed ultra-sensitive assays. Limitations include low detection rates, heterogeneous methodologies, small sample sizes, and predominantly exploratory study designs. Conclusions and Clinical Implications: Currently, its most promising application is not broad screening, but as a selective, biology-driven tool for detecting minimal residual disease and refining risk assessment. CtDNA acts as a biological risk modifier, potentially guiding the escalation or de-escalation of adjuvant therapy. However, prospective biomarker-driven trials are required to validate these strategies before routine clinical implementation.

## Simple Summary

This review highlights the limited clinical utility of circulating tumor cells (CTCs) and the emerging prognostic relevance of circulating tumor DNA (ctDNA) in non-metastatic prostate cancer. CtDNA may serve as a marker of minimal residual disease and biological aggressiveness, pending validation in prospective trials.

## 1. Introduction

Prostate cancer represents one of the most frequently diagnosed malignancies in men worldwide and displays marked biological and clinical heterogeneity. While the majority of patients are diagnosed with non-metastatic disease and can be treated with curative intent, a clinically relevant proportion will ultimately experience biochemical recurrence and disease progression despite apparently localized presentation. Current risk stratification relies on serum PSA, Gleason score, and clinical or pathological staging, which are imperfect surrogates of the underlying tumor biology. Consequently, there is an unmet need for minimally invasive biomarkers capable of capturing tumor heterogeneity, identifying aggressive disease early, and refining prognosis beyond conventional clinicopathological parameters [1].

Liquid biopsy has emerged as a transformative concept in oncology, defined as the analysis of tumor-derived material released into body fluids, most commonly blood but also urine or other biofluids. Liquid biopsy encompasses multiple analytes, including circulating tumor cells (CTCs), circulating tumor DNA (ctDNA) within the broader pool of cell-free DNA (cfDNA), extracellular vesicles and exosomes, and tumor-associated RNA species such as microRNAs. Unlike tissue biopsy, liquid biopsy offers a minimally invasive, repeatable approach that may reflect spatial and temporal tumor heterogeneity and enable longitudinal monitoring [2,3,4,5,6,7,8].

Recent reviews have highlighted the rapid technological evolution of liquid biopsy and its expanding role across genitourinary malignancies [9,10]. These reviews emphasize that, while ctDNA is now firmly established as a prognostic and predictive biomarker in metastatic prostate cancer, its application in non-metastatic disease remains exploratory [9,10]. Another recent review focused on minimal residual disease (MRD) to further underscore the potential of ultra-sensitive, tumor-informed ctDNA assays to detect subclinical disease after definitive local therapy, a concept that may be particularly relevant in localized prostate cancer, where conventional imaging and PSA lack sensitivity. Once more, evidence on prostate cancer in the setting of MRD is limited [11].

The present overview aims to comprehensively summarize the current state-of-the-art on liquid biopsy in non-metastatic prostate cancer, with a focus on ctDNA and CTC-based approaches. We synthesize data from original studies and reviews, describe methodological heterogeneity, discuss biological and technical limitations, and outline future perspectives, highlighting the substantial knowledge gap that persists in contrast to the metastatic setting.

## 2. Materials and Methods

Given the emerging and heterogeneous nature of liquid biopsy research in non-metastatic prostate cancer, we designed a systematic literature search following the principles of the Preferred Reporting Items for Systematic Reviews and Meta-Analyses (PRISMA). Although the available evidence base is limited and characterized by methodological diversity that precludes formal meta-analysis, a structured and transparent search strategy was deemed appropriate to ensure comprehensive coverage and reproducibility.

Electronic searches were performed in PubMed/MEDLINE, the Cochrane Library, Web of Science, and Google Scholar. The search strategy combined controlled vocabulary and free-text terms related to prostate cancer and liquid biopsy, including combinations of “prostate cancer,” “localized,” “non-metastatic,” “liquid biopsy,” “circulating tumor DNA,” “ctDNA,” “cell-free DNA,” “circulating tumor cells,” “CTC,” “exosomes,” and “minimal residual disease.” Searches were conducted without initial date restrictions and were last updated in 2025. Reference lists of relevant review articles and original studies were manually screened to identify additional eligible publications.

We included prospective and retrospective original studies evaluating liquid biopsy approaches in patients with non-metastatic or clinically localized prostate cancer, defined as the absence of radiologically evident distant metastases at diagnosis. Conference abstracts from major oncology and urology meetings were considered when sufficient methodological and outcome data were available, particularly for MRD-focused studies. Review articles were used to contextualize findings, but were not included in the quantitative synthesis of results.

Studies exclusively enrolling metastatic or castration-resistant prostate cancer populations were excluded unless data for a clearly defined non-metastatic subgroup could be extracted. Case reports, preclinical studies, and purely technical validation studies without clinical outcomes were also excluded. Two reviewers independently screened titles and abstracts, followed by full-text assessment of potentially eligible studies. Discrepancies were resolved by consensus.

Risk of bias was assessed qualitatively, considering study design, patient selection, sample size, timing of liquid biopsy sampling, analytical validity of the assays, and completeness of outcome reporting. Given the observational nature of most included studies and the absence of randomized comparisons, both the Newcastle–Ottawa Scale and MINORS scale were used to assess adequate risk of bias in case of small, single-group, pilot studies. The results are shown in Table 1 and Table 2.

## 3. Results

The evidence base evaluating liquid biopsy in non-metastatic prostate cancer is limited but evolving. A total of eleven PubMed-indexed original studies met criteria for inclusion and are summarized in Table 3. These studies collectively included approximately 850 patients with clinically localized and non-metastatic prostate cancer, the majority treated with radical prostatectomy. Study designs were predominantly exploratory and observational, with substantial heterogeneity in liquid biopsy analytes, analytical platforms, sampling time points, and clinical endpoints. Other features and details are reported in Supplementary Table S1.

Early studies focused on circulating tumor cells (CTCs) and consistently demonstrated low detection rates in localized and biochemical recurrence settings. In a small prospective pilot study, Khurana et al. [12] detected CTCs using the CellSearch system in only 1 of 10 evaluable patients undergoing radical prostatectomy, highlighting limited sensitivity in localized disease. In a larger cohort, Meyer et al. [15] similarly reported infrequent preoperative CTC detection (11%) and found no association with clinicopathologic features or biochemical recurrence-free survival after a median follow-up of 48 months. Pal et al. [13] achieved higher preoperative CTC detection rates (49%) in a selected high-risk cohort using a modified CellSearch approach; however, intact CTC counts were not prognostic, although specific CTC fragments expressing CD133 or altered E-cadherin were associated with early biochemical recurrence. In the biochemical recurrence setting, Aragon-Ching et al. [14] and Grisanti et al. [16] reported very low CTC detection rates (8.3% and 3%, respectively), with limited prognostic value and absent detection even in patients who later developed metastatic disease. Collectively, these studies indicate that EpCAM-based CTC assays have limited sensitivity and clinical utility in non-metastatic prostate cancer.

The introduction of next-generation sequencing enabled further investigation of circulating tumor DNA.

The study by Hennigan et al. [17] investigated the detectability and clinical relevance of circulating tumor DNA (ctDNA) in men with clinically localized prostate cancer undergoing radical prostatectomy. This prospective observational study enrolled approximately 100 patients with localized disease, alongside comparator cohorts of patients with metastatic prostate cancer and healthy controls. Peripheral blood plasma was collected preoperatively and, in a subset of patients, postoperatively. An ultra-deep targeted next-generation sequencing assay using a prostate cancer-specific gene panel was employed to detect tumor-derived somatic mutations previously identified in matched tumor tissue, maximizing sensitivity for low-level ctDNA detection. CtDNA positivity was defined by the presence of tumor-specific variants above assay-specific background error rates rather than a fixed numeric cutoff. The primary endpoint was ctDNA prevalence and abundance in localized versus metastatic disease, with secondary analyses assessing associations with adverse pathological features. CtDNA was rarely detectable and present at extremely low variant allele fractions in localized prostate cancer, in contrast to metastatic cases, indicating minimal tumor DNA shedding at early disease stages. Limitations included the low ctDNA detection rate precluding outcome correlations, reliance on highly specialized sequencing platforms, and the possibility that even ultra-deep sequencing remains insufficiently sensitive for minimal residual disease (MRD) detection. The authors highlighted the biological challenge of limited ctDNA shedding in early-stage disease and the need for more sensitive analytical approaches.

Lau et al. [18] similarly evaluated ctDNA detectability and prognostic significance in patients with localized prostate cancer treated with radical prostatectomy using two complementary cohorts. In a small prospective cohort, paired tumor and germline whole-genome sequencing identified patient-specific somatic alterations, which were interrogated in plasma collected preoperatively and postoperatively using bespoke ultra-deep sequencing with unique molecular identifiers. In a larger retrospective cohort of 189 patients enriched for high-risk disease, preoperative plasma samples were analyzed using tagged-amplicon deep sequencing (TAm-Seq) targeting TP53. CtDNA positivity was defined using stringent assay-specific thresholds rather than a numeric cutoff. The primary endpoint was the association between preoperative ctDNA detection and clinical outcomes, including biochemical recurrence-free and metastasis-free survival. CtDNA was detected in 2 of 8 patients in the personalized cohort and in 22 of 189 patients (12%) in the TP53 cohort, and in both analyses ctDNA positivity was associated with aggressive disease biology and significantly shorter metastasis-free survival. Limitations included low detection rates, potential confounding from clonal hematopoiesis in the TP53 analysis, reliance on specialized sequencing platforms, and enrichment for high-risk disease. Nonetheless, the study provided proof-of-concept that ctDNA detection, despite low prevalence, may have strong prognostic significance in localized prostate cancer.

Pope et al. [19] further advanced the field by applying the ultra-sensitive INVAR methodology in a multicenter cohort of 118 patients with localized prostate cancer undergoing radical prostatectomy. Preoperative plasma samples were collected, with additional plasma from prostate cancer–free controls. Whole-genome sequencing of primary tumor tissue was used to identify patient-specific mutations, which were interrogated in plasma using a custom targeted sequencing approach coupled with the INVAR method, allowing aggregation of signal across thousands of tumor-informed loci. CtDNA burden was quantified as an integrated mutant allele fraction (IMAF), with ctDNA positivity defined using patient-specific thresholds to achieve 95% specificity. Primary endpoints were biochemical recurrence-free and metastasis-free survival. CtDNA was detectable in 16% of patients and was significantly associated with adverse clinicopathologic features and shorter biochemical recurrence-free and metastasis-free survival, independent of established prognostic variables. Limitations included enrichment for high-risk disease, a limited number of metastatic events, and the requirement for tumor whole-genome sequencing, which currently limits scalability. Overall, this study demonstrated that tumor-informed ultrasensitive ctDNA detection is feasible in localized prostate cancer and identifies a biologically aggressive subgroup at increased risk of postoperative recurrence.

The retrospective real-world study by Fei et al. [20] evaluated the prognostic value of preoperative ctDNA detection in patients with non-metastatic prostate cancer undergoing definitive local therapy. Among 161 treatment-naïve patients, 139 had available preoperative plasma samples and 31 had tumor tissue for genomic profiling. Targeted next-generation sequencing was used to identify somatic alterations, and ctDNA burden was estimated using mutant allele fractions corrected for diploid genomes. CtDNA positivity was defined as any detectable ctDNA fraction greater than 0%. The primary endpoint was biochemical recurrence following radical prostatectomy. CtDNA was detectable preoperatively in 65.5% of patients and was strongly associated with shorter biochemical progression-free survival, remaining an independent predictor in multivariable analyses. Limitations included the retrospective design, heterogeneity in treatments and sequencing platforms, lack of serial postoperative sampling, and potential selection bias. Despite these limitations, the study suggests that early preoperative ctDNA detection may identify patients with biologically aggressive non-metastatic disease at high risk of recurrence.

Zang et al. [21] explored ctDNA for MRD detection using enriched amplicon sequencing with unique molecular identifiers in a prospective feasibility study of 11 patients with localized prostate cancer undergoing radical prostatectomy, most with high-grade tumors. Plasma samples were collected preoperatively, immediately postoperatively, and at one month postoperatively. A tumor-informed approach incorporating tumor and germline exome sequencing was used to identify founder mutations, followed by highly sensitive plasma DNA analysis. CtDNA detection was defined by tumor-specific variants above background error without a fixed cutoff. CtDNA was detectable in 45% of patients preoperatively and persisted in over 60% postoperatively, with median tumor fractions below 0.1%. Persistent ctDNA at one month appeared associated with later biochemical recurrence. Limitations included the very small sample size, exploratory design, lack of standardized postoperative management, and absence of predefined outcome-linked thresholds. Nonetheless, the study demonstrated the technical feasibility of tumor-informed enriched amplicon ctDNA sequencing for MRD detection and supports further validation.

Finally, Kluge et al. [22] compared ctDNA analysis with [^68^Ga]Ga-PSMA-11 PET/CT imaging in a retrospective cross-sectional study of 130 men across the prostate cancer disease spectrum. Plasma-derived cfDNA was analyzed using low-pass whole-genome sequencing to quantify ctDNA via copy number profiling. CtDNA positivity was defined by statistically significant copy number alterations, while PSMA PET positivity was based on qualitative and quantitative imaging parameters. PSMA PET showed substantially higher detection rates than ctDNA, particularly in localized and low-volume disease, whereas ctDNA detection was more frequent in metastatic and castration-resistant cases. Both elevated ctDNA levels and high PSMA tumor volume were independently associated with inferior overall survival. Limitations included the retrospective design, cohort heterogeneity, short follow-up, and sensitivity constraints of low-pass sequencing in low tumor burden settings. Despite these limitations, the study highlights the superior diagnostic sensitivity of PSMA PET and supports a complementary prognostic role for ctDNA, particularly in advanced disease.

## 4. Discussion

The current overview collectively illustrates the evolution of liquid biopsy research in non-metastatic prostate cancer, progressing from early circulating tumor cell–based approaches to highly sensitive next-generation sequencing strategies for circulating tumor DNA detection. This trajectory reflects both increasing technical sophistication and a growing understanding of the biological constraints inherent to localized disease.

Initial investigations focused on circulating tumor cells [2,3,4,5,6,7,8], largely extrapolating methodologies validated in metastatic prostate cancer. As shown by Khurana et al., EpCAM-based CTC enrichment using the CellSearch platform yielded extremely low detection rates in patients with clinically localized prostate cancer. These findings were consistent across studies despite the inclusion of intermediate- and high-risk patients and underscore fundamental biological limitations, including minimal tumor cell dissemination prior to metastatic spread. In addition, epithelial marker–dependent capture systems may fail to detect phenotypically heterogeneous or epithelial-to-mesenchymal transition-like tumor cells. Collectively, these limitations explain why CTC enumeration has not demonstrated clinical utility in the non-metastatic setting and has largely been abandoned in favor of alternative liquid biopsy analytes [4].

The advent of next-generation sequencing enabled the investigation of ctDNA [9,10], initially through tumor-naïve approaches. Hennigan et al. provided critical biological insight by demonstrating very low tumor fractions in preoperative plasma from patients with localized prostate cancer. These findings confirmed that limited tumor DNA shedding represents a major barrier to ctDNA detection in early-stage disease and highlighted the need for ultra-sensitive analytical techniques. Importantly, this study helped reframe negative ctDNA findings not as a technical failure but as a reflection of disease biology.

Tumor-informed ctDNA strategies marked a pivotal advance. Lau et al. [18] were among the first to apply personalized sequencing approaches in clinically localized prostate cancer, demonstrating that ctDNA detection, while rare, identified patients with highly aggressive disease and rapid progression. Their subsequent larger analysis showed that detection of TP53 mutations in preoperative plasma was associated with significantly shorter metastasis-free survival, providing robust evidence that ctDNA carries clinically meaningful prognostic information even when present at very low levels. These studies established proof-of-concept that ctDNA can capture aggressive tumor biology beyond conventional clinicopathological risk stratification.

The most compelling evidence to date derives from the INVAR study by Pope et al. [19] (Table 1), which employed an ultra-sensitive, tumor-informed sequencing approach integrating thousands of patient-specific mutations. Using this methodology, ctDNA was detectable in approximately 16% of patients with localized disease, with mutant allele fractions several orders of magnitude lower than those typically observed in metastatic prostate cancer. Importantly, ctDNA positivity independently predicted biochemical recurrence and metastasis-free survival. This study demonstrated that sensitivity, rather than biological irrelevance, is the principal limitation of ctDNA analysis in non-metastatic prostate cancer and establishes a benchmark for future assay development.

Tumor-naïve NGS approaches, such as that reported by Fei et al. [20], offer a complementary perspective. In this study, ctDNA was detectable in a higher proportion of patients and independently associated with biochemical recurrence (Table 1). However, broader genomic coverage and less stringent analytical thresholds may increase detection rates at the potential cost of reduced specificity. These findings highlight a fundamental trade-off between sensitivity, specificity, and scalability that will need to be addressed before widespread clinical implementation.

Beyond preoperative risk stratification, ctDNA-based detection of minimal residual disease represents a particularly promising application. Zang et al. [21] demonstrated the feasibility of detecting ctDNA after radical prostatectomy using enriched amplicon sequencing with unique molecular identifiers, with persistent ctDNA detection associated with subsequent PSA relapse. Although limited by a small sample size, this study supports the concept that ctDNA may detect molecular recurrence earlier than PSA and could inform postoperative management strategies, including adjuvant or early salvage therapy.

Integration with advanced imaging further supports the biological relevance of ctDNA in non-metastatic disease. Kluge et al. [22] demonstrated a correlation between ctDNA levels and PSMA PET tumor burden, even in patients without overt metastases. These findings suggest that ctDNA reflects true disease burden and may provide complementary information to imaging, particularly in the context of microscopic residual disease.

Overall, the accumulated evidence highlights a difference in the maturity and clinical applicability of liquid biopsy between metastatic and non-metastatic prostate cancer [22,23,24,25,26,27]. In metastatic disease, liquid biopsy—particularly ctDNA analysis—has transitioned from an exploratory biomarker to an integral component of routine clinical care. As recently summarized in contemporary state-of-the-art reviews, ctDNA testing in metastatic prostate cancer is now routinely used for predictive biomarker assessment, including homologous recombination repair gene alterations, microsatellite instability, tumor mutational burden, and androgen receptor pathway alterations, with multiple FDA-approved therapies explicitly allowing or requiring liquid biopsy–based patient selection. In this setting, higher tumor burden, increased cellular turnover, and widespread dissemination result in robust ctDNA shedding, enabling reliable detection with commercially available platforms and facilitating longitudinal disease monitoring.

In contrast, liquid biopsy in non-metastatic prostate cancer is still under investigation, with biological and technical issues. Several limitations remain consistent across studies. Most investigations are exploratory, single-center, and underpowered for definitive clinical conclusions. There is marked heterogeneity in assay platforms, genomic targets, analytical pipelines, cut-off definitions, and timing of blood sampling. Tumor-informed approaches, while analytically superior, require access to high-quality tumor tissue and complex bioinformatic workflows, potentially limiting scalability and cost-effectiveness. Conversely, tumor-naïve approaches may be more practical but require careful validation to ensure clinical specificity [9].

In localized prostate cancer, the most realistic and clinically meaningful future perspective for liquid biopsy is not broad disease detection or routine upfront risk stratification, but rather selective, biology-driven applications that address unmet needs left by PSA, imaging, and pathology. The field is converging toward a few clearly defined directions. Exploring gene expression inference from cfDNA is both a logical and necessary next step. Our review highlights that we are moving away from simple CTC toward high-sensitivity molecular profiling; if we could aggregate enough signal, we would identify biologically aggressive subgroups that conventional tools miss. Further investigation is warranted to see if gene expression can provide a more nuanced ‘biological risk modifier’ to improve outcomes and reduce overtreatment. Our review indicates that by ‘aggregating signal’ we can overcome this sensitivity barrier. This approach warrants investigation because it fits the ideal role of liquid biopsy in this setting: not as a broad screening tool, but as a selective, biology-driven instrument. It has the potential to act as a biological risk modifier, allowing clinicians to distinguish aggressive disease from indolent cases more effectively than PSA or pathology alone. Table 4 summarizes the clinical implementation pathways. Despite tumor-informed ctDNA assays offering a compelling framework for MRD detection in nmPC, their use is constrained by cost, scalability, and regulation [28]. Cost-effectiveness of MRD testing is only justifiable if MRD-guided therapy convincingly improves survival or avoids ineffective treatment. Until prospective trials show that MRD-adapted adjuvant or salvage strategies in nmPCa translate into clinically meaningful benefit, the reimbursement for these high-complexity assays is likely to remain restricted to research or selected high-risk scenarios. Scalability is another major barrier. Tumor-informed MRD workflows depend on access to high-quality FFPE tissue, extraction of matched tumor and germline DNA, and bioinformatic pipelines capable of designing and validating panels for each patient, often at sequencing depths > 100,000 × across dozens to hundreds of loci [28]. The personalized design of tumor-informed MRD and the ultra-deep sequencing impose a high fixed workload and may favor centralization in a limited number of specialized laboratories, limiting diffusion into community practice. Low ctDNA shedding in nmPCa also forces laboratories to balance very stringent limits of detection against the competing risk of false positives from clonal hematopoiesis, requiring matched-normal sequencing and sophisticated error-suppression strategies [28]. Moreover, routine implementation would require harmonized pre-analytical procedures (tubes, processing times, storage), standardized sampling schedules around surgery or radiotherapy, and informatics systems that can integrate tumor sequencing, longitudinal plasma results and decision algorithms—capabilities that are not yet uniformly available in urology or radiation oncology workflows [29]. Regulatory frameworks are evolving, but add further complexity. Most tumor-informed ctDNA MRD assays currently operate as high-complexity laboratory-developed tests; new consensus protocols from the BLOODPAC consortium now define generic standards for analytical validation (limit of detection, precision, specificity) tailored to tumor-informed MRD platforms [30]. The US FDA’s 2024 final guidance on ctDNA in early-stage solid-tumor drug development specifies expectations for assay calibration, longitudinal sampling and clinical validation when ctDNA-based MRD is used as an enrichment biomarker or surrogate endpoint in trials (U.S. Food and Drug Administration). In the European Union, full application of the IVDR (EU 2017/746) [31] has raised the evidentiary bar for ctDNA IVDs, mandating robust analytical and clinical performance data and CE-marking for commercial assays, with substantial implications for both manufacturers and diagnostic laboratories [32]. Overall, in non-metastatic prostate cancer—where the evidence base for tumor-informed MRD is still less mature than in colorectal or breast cancer—current expert reviews position ctDNA primarily as a promising research and risk-stratification tool rather than a standard-of-care test. Broad clinical adoption will ultimately depend on demonstrating that the added cost and operational complexity of tumor-informed MRD are justified by clear improvements in patient outcomes and on harmonizing these assays with emerging regulatory and reimbursement frameworks.

The clearest and most clinically relevant application is MRD detection after curative-intent therapy, where tumor-informed ctDNA assays can identify a small subset of patients at very high risk of early recurrence and metastasis before biochemical relapse becomes evident. This creates an opportunity for ctDNA-guided treatment personalization, enabling escalation of adjuvant therapy in ctDNA-positive patients while supporting de-escalation or omission of therapy in ctDNA-negative individuals. Beyond MRD, ctDNA may serve as a biological risk modifier in selected high-risk patients, refining prognostication beyond conventional clinicopathologic variables when integrated into multivariable models. Longitudinal ctDNA monitoring represents an additional emerging use, offering dynamic insights into molecular relapse and clonal evolution during adjuvant or salvage treatment. Ultimately, the clinical impact of liquid biopsy in localized prostate cancer will depend on prospective, biomarker-driven trials demonstrating that ctDNA-guided strategies meaningfully improve outcomes and reduce overtreatment, positioning liquid biopsy as a complementary tool rather than a replacement for existing standards.

## 5. Conclusions

The current evidences support a clear paradigm shift in non-metastatic prostate cancer liquid biopsy research, moving away from CTC enumeration toward ultra-sensitive ctDNA sequencing. While ctDNA detection rates remain low due to biological constraints, detection consistently identifies patients with aggressive disease and poor outcomes. Minimal residual disease detection may emerge as the most clinically promising application, but robust prospective validation and assay standardization are essential before liquid biopsy can be routinely integrated into clinical decision-making for non-metastatic prostate cancer.

## Figures and Tables

**Table 1:** Risk of Bias assessment according to MINORS score.

									Additional Criteria in Case of Comparative Studies			
	A Clearly Stated Aim	Inclusion of Consecutive Patients	Prospective Collection of Data	End-Points Appropriate to the Aim of the Study	Unbiased Assessment of Study Endpoint	Follow-Up Period Appropriate to the Aim of the Study	Loss to Follow-Up Less than 5%	Prospective Calculation of the Study Size	Adequate Control Group	Contemporary Groups	Baseline Equivalence of Groups	Adequate Statistical Analyses
Khurana et al., 2013 [12]	2	1	2	2	2	2	1	1				
Pal et al., 2015 [13]	2	1	2	2	1	1	2	0				
Aragon-Ching et al., 2015 [14]	2	2	2	2	1	2	2	2				
Meyer et al., 2016 [15]	2	2	2	2	1	2	0	0				
Grisanti et al., 2016 [16]	2	2	2	2	1	1	2	2				
Hennigan et al., 2019 [17]	2	2	2	2	2	2	2	0	2	2	1	2
Lau et al., 2020 [18]	2	1	2	2	2	2	0	0	2	2	0	1
Pope et al., 2024 [19]	2	2	2	2	2	2	2	0	2	2	1	2
Fei et al., 2023 [20]	2	2	1	2	2	1	2	0	2	2	1	2
Zang et al., 2025 [21]	2	1	2	2	1	2	1	0				
Kluge et al., 2024 [22]	2	2	2	2	2	2	2	0	2	2	2	2

**Table 2:** Risk of Bias assessment according to the Newcastle–Ottawa scale.

Study	Selection (Max 4★)	Comparability (Max 2★)	Outcome (Max 3★)	Quality	Points
Khurana et al., 2013 [12]	★		★★	Low	3
Pal et al., 2015 [13]	★		★★	Low	3
Aragon-Ching et al., 2015 [14]	★		★★	Low	3
Meyer et al., 2016 [15]	★★★★	★★	★★★	High	9
Grisanti et al., 2016 [16]	★		★★	Low	3
Hennigan et al., 2019 [17]	★★		★★	Low	4
Lau et al., 2020 [18]	★★★★	★★	★★★	High	9
Pope et al., 2024 [19]	★★★★	★★	★★★	High	9
Fei et al., 2023 [20]	★★★★	★★	★★★	High	9
Zang et al., 2025 [21]	★★		★★	High	4
Kluge et al., 2024 [22]	★★★	★★	★★	Moderate	7

Selection (0–4 stars): representativeness of cohort/cases, selection of controls, exposure/outcome definition; Comparability (0–2 stars): control for confounders; Outcome/Exposure (0–3 stars): assessment method, follow-up adequacy, data completeness. Higher score = lower risk of bias.

**Table 3:** Key studies evaluating CTCs and ctDNA liquid biopsy approaches in non-metastatic prostate cancer.

Study	Design	Population (n)	Analyte	Assay/Platform	Specimen and Timing	Definition of Positivity (Cut-Off)	Primary Endpoint and Main Findings	Key Limitations
Khurana et al. 2013 [12]	Prospective pilot	10 localized PCa	CTCs	CellSearch (EpCAM-based immunomagnetic enrichment)	Peripheral blood, preoperative	≥1 CTC/7.5 mL blood	Prevalence of CTCs; CTCs are rare, only 1/10 pts is CTC positive	Very small sample size; single-center; no outcome correlation; EpCAM dependence
Pal et al. 2015 [13]	Prospective observational	35 high-risk localized PCa undergoing RP	CTCs and CTC fragments	Modified CellSearch (Ficoll enrichment, large-volume blood, open channel phenotyping)	Peripheral blood, pre- and post-RP	≥1 CTC/7.5 mL blood	BCR; no correlation between CTC count and BCR but association with CD133 and E-Cadherin positive CTC	High preoperative CTC detection rate (49%); intact CTC counts not prognostic; CD133- and E-cadherin–positive CTC fragments associated with 1-yr BCR; small cohort; complex methodology
Aragon-Ching et al. 2015 [14]	Prospective observational	36	CTCs	CellSearch (EpCAM-based)	Peripheral blood, at study entry	≥1 CTC/7.5 mL blood	PSA kinetics; very low CTC detection rate (8.3%); CTC positivity associated with rapid PSADT and occult metastases;	Limited sensitivity; heterogeneous cohort
Meyer et al. 2016 [15]	Retrospective observational	152 localized PCa undergoing RP	CTCs	CellSearch (EpCAM-based immunomagnetic enrichment)	Peripheral blood, preoperative	≥1 CTC/7.5 mL blood	Early BCR; low CTC detection rate; no significant prognostic value	EpCAM-dependent assay; limited biological shedding in localized disease
Grisanti et al. 2016 [16]	Prospective observational	29 after RP and radiation	CTCs post-op	CellSearch (EpCAM-based)	Peripheral blood, at PSA recurrence	≥1 CTC/7.5 mL blood	CTC detection at BCR; CTCs are detected in a minority of patients at very low levels	Extremely low detection rate (3%); no association with PSA kinetics or progression; limited sensitivity in the MRD setting; EpCAM-dependent assay
Hennigan et al. 2019 [17]	Prospective observational	~112 localized PCa (+metastatic and controls)	ctDNA	Ultra-deep targeted NGS (tumor-informed in 9)	Plasma, preoperative ± postoperative	Tumor-specific variants above background error (no fixed numeric cutoff)	Detectability of ctDNA; ultra-low-pass whole-genome sequencing and targeted resequencing did not detect ctDNA in plasma acquired before surgery or before recurrence.	Extremely low detection rate; limited outcome associations; technical complexity
Lau et al. 2020 [18]	Prospective + retrospective cohorts	8 (tumor-informed) + 189 (TP53 TAm-Seq)	ctDNA pre-op in TP53 TAm-Seq; post-op in tumor-informed (8)	Personalized ultra-deep NGS; TP53 TAm-Seq	Plasma, preoperative; subset post-op (24 h, 6 weeks)	Tumor-specific variants exceeding stringent error thresholds	Metastasis-free survival in TP53 TAm-Seq; Tumor variants in ctDNA were identified pre-op in 2/8 patients, which in both cases remained detectable post-op. Patients with tumor variants in ctDNA had extremely rapid disease recurrence and progression.	Low sensitivity; clonal hematopoiesis risk; high-risk enrichment
Pope et al. 2024 [19]	Multicenter observational	118 localized PCa (+27 controls)	ctDNA pre-op	Tumor-informed INVAR (WGS-derived)	Plasma, preoperative	Patient-specific threshold achieving 95% specificity (IMAF)	BCR-free and metastasis-free survival	High cost; need for tumor WGS; limited scalability
Fei et al. 2023 [20]	Retrospective real-world	161 non-metastatic PCa (139 plasma)	ctDNA pre-op	NGS (tumor-naïve in 130; 9 matched biopsied tumor and peripheral sample)	Plasma, preoperative only	Any detectable ctDNA (>0%)	BCR; pre-op ctDNA detection could identify pat at high risk of recurrence	Retrospective design; platform heterogeneity; no post-op MRD assessment
Zang et al. 2025 [21]	Prospective feasibility	11 localized PCa	ctDNA pre- and post-op	Tumor-informed enriched amplicon sequencing with UMIs	Plasma, pre-op, immediate post-op, 1 month post-op	Tumor-specific variants above background (no fixed cutoff)	Minimal residual disease (MRD); residual ctDNA detection is associated with relapse	Very small cohort; exploratory; non-powered
Kluge et al. 2024 [22]	Retrospective cross-sectional	130 PCa (all stages)	ctDNA	Low-pass whole-genome sequencing (CNV-based)	Plasma, single time point	Significant CNVs above noise	Detection rate: Correlations between ctDNA concentrations and PSMA-positive tumor volume were significant in all and castration-resistant; however, not in hormone- sensitive pat. PSMA-TV and ctDNA levels were associated with survival outcomes	

**Table 4:** Possible clinical implementation pathways.

Clinical Implementation Pathway	Surgery	MRD	Adjuvant	MRD Monitoring	First Line	Monitoring	Progressive Disease
Tumor-informed Liquid Biopsy		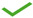 Tissue required	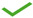	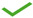 Limitation: treatment pressure and molecular selection	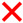 treatment pressure and molecular selection	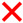 treatment pressure and molecular selection	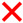 treatment pressure and molecular selection
Tumor agnostic Liquid Biopsy		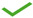	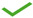	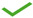	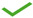	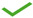	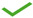
dPCR (cheaper, faster)		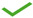 Tissue required	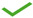	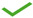 Limitation: treatment pressure and molecular selection	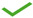 Limitation: treatment pressure and molecular selection	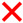 treatment pressure and molecular selection	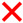 treatment pressure and molecular selection
NGS (expensive, labor-intensive)		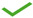	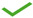	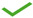	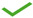	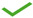	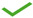
Timepoints		1 month after surgery; every 3 months	Baseline	Every 3 months	Baseline	Every 3 months	At PD
Risk of false negatives		High	High	High	Medium	Medium	Medium-Low
Tumor Fraction evaluation		Informative: False negatives CHIP Germ-line variants	Informative: False negatives CHIP Germ-line variants	Informative: False negatives CHIP Germ-line variants	Informative: False negatives CHIP Germ-line variants	Informative: False negatives CHIP Germ-line variants	Informative: False negatives CHIP Germ-line variants

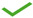
 stands for applicable; 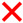 stands for not applicable.

## Data Availability

No new data were created or analyzed in this study. Data sharing is not applicable to this article.

## Supplementary Materials

The following supporting information can be downloaded at: https://www.mdpi.com/article/10.3390/cancers18050800/s1, Table S1: Features and details.

## References

[1] Sighinolfi M.C., Pallotta G., Assumma S., Panio E., Pinto F., Gavi F., Totaro A., Presutti S., Pasciuto T., Nero C. (2025). A novel comprehensive cancer genome profiling for non-metastatic prostate cancer: Study protocol with FPG500 to detect actionable alterations representative of progressive disease. BJU Int..

[2] Rzhevskiy A.S., Kapitannikova A.Y., Butnaru D.V., Shpot E.V., Joosse S.A., Zvyagin A.V., Warkiani M.E. (2022). Liquid biopsy in diagnosis and prognosis of non-metastatic prostate cancer. Biomedicines.

[3] Dathathri E., Isebia K.T., Abali F., Lolkema M.P., Martens J.W.M., Terstappen L.W.M.M., Bansal R. (2022). Liquid biopsy based circulating biomarkers in metastatic prostate cancer. Front. Oncol..

[4] Andree K.C., van Dalum G., Terstappen L.W.M.M. (2016). Challenges in circulating tumor cell detection by the CellSearch system. Mol. Oncol..

[5] Lu Y.T., Delijani K., Mecum A., Goldkorn A. (2019). Current status of liquid biopsies for the detection and management of prostate cancer. Cancer Manag. Res..

[6] Trujillo B., Wu A., Wetterskog D., Attard G. (2022). Blood-based liquid biopsies for prostate cancer: Clinical opportunities and challenges. Br. J. Cancer.

[7] Zainfeld D., Goldkorn A. (2018). Liquid biopsy in prostate cancer: Circulating tumor cells and beyond. Cancer Treat. Res..

[8] Casanova-Salas I., Athié A., Boutros P.C., Del Re M., Miyamoto D.T., Pienta K.J., Posadas E.M., Sowalsky A.G., Stenzl A., Wyatt A.W. (2021). Quantitative and qualitative analysis of blood-based liquid biopsies to inform clinical decision-making in prostate cancer. Eur. Urol..

[9] Maughan B.L., Dyrskjøt L., Grivas P., Alcaraz A., Antonarakis E.S., Bilen M.A., Bivalacqua T., Cooperberg M.R., Cheng L., Gupta S. (2025). The role of liquid biopsy in the management of patients with genitourinary malignancies. Eur. Urol..

[10] Chanhih N., Laraqui A., Hassine S., Ameur A., Hamedoun L., El Annaz H., Abi R., Tagajdid M.R., Amine I.L., Ennibi K. (2025). Circulating tumor DNA as a biomarker for precision medicine in prostate cancer: A systematic review. Int. J. Mol. Sci..

[11] Barata P.C., Zarrabi K.K., Bex A., Grivas P., Hermann K., Hofman M.S., Li R., Lopez-Beltran A., Padani A.R., Powles T. (2025). Novel methods to assess tumor burden and minimal residual disease in genitourinary cancers. Eur. Urol..

[12] Khurana K.K., Grane R., Borden E.C., Klein E.A. (2013). Prevalence of circulating tumor cells in localized prostate cancer. Curr. Urol..

[13] Pal S.K., He M., Wilson T., Liu X., Zhang K., Carmichael C., Torres A., Hernandez S., Lau C., Agarwal N. (2015). Detection and phenotyping of circulating tumor cells in high-risk localized prostate cancer. Clin. Genitourin. Cancer.

[14] Aragon-Ching J.B., Siegel R.S., Frazier H., Andrawis R., Hendricks F., Phillips M., Jarrett T., Guebre-Xabiher H., Patierno S., Simmens S.J. (2015). Circulating tumor cells in biochemical recurrence of prostate cancer. Clin. Genitourin. Cancer.

[15] Meyer C.P., Pantel K., Tennstedt P., Stroelin P., Schlomm T., Heinzer H., Riethdorf S., Steuber T. (2016). Limited prognostic value of preoperative circulating tumor cells for early biochemical recurrence in patients with localized prostate cancer. Urol. Oncol..

[16] Grisanti S., Antonelli A., Buglione M., Almici C., Foroni C., Sodano M., Triggiani L., Greco D., Palumbo C., Marini M. (2016). Analysis of circulating tumor cells in prostate cancer patients at PSA recurrence and review of the literature. Anticancer Res..

[17] Hennigan S.T., Trostel S.Y., Terrigino N.T., Voznesensky O.S., Schaefer R.J., Whitlock N.C., Wilkinson S., Carrabba N.V., Atway R., Shema S. (2019). Low abundance of circulating tumor DNA in localized prostate cancer. JCO Precis. Oncol..

[18] Lau E., McCoy P., Reeves F., Chow K., Clarkson M., Kwan E.M., Packwood K., Northen H., He M., Kingsbury Z. (2020). Detection of ctDNA in plasma of patients with clinically localised prostate cancer is associated with rapid disease progression. Genome Med..

[19] Pope B., Park G., Lau E., Belic J., Lach R., George A., McCoy P., Nguyen A., Grima C., Campbell B. (2024). Ultrasensitive detection of circulating tumour DNA enriches for patients with a greater risk of recurrence of clinically localised prostate cancer. Eur. Urol..

[20] Fei X., Du X., Gong Y., Liu J., Fan L., Wang J., Wang Y., Zhu Y., Pan J., Dong B. (2023). Early plasma circulating tumor DNA as a potential biomarker of disease recurrence in non-metastatic prostate cancer. Cancer Res. Treat..

[21] Zang P.D., Lama D., Huang Z., Jaime-Casas S., Contente-Cuomo T., Stampar M., Marshall A., Dinwiddie D., Berens M.E., Pond S. (2025). Feasibility of enriched amplicon circulating tumor DNA sequencing to detect minimal residual disease after prostatectomy in localized prostate cancer. J. Clin. Oncol..

[22] Kluge K., Einspieler H., Haberl D., Spielvogel C., Amereller D., Egger G., Kramer G., Grubmüller B., Shariat S., Hacker M. (2024). Comparison of discovery rates and prognostic utility of [^68^Ga]Ga-PSMA-11 PET/CT and circulating tumor DNA in prostate cancer—A cross-sectional study. Eur. J. Nucl. Med. Mol. Imaging.

[23] Ravindranathan D., Russler G.A., Yantorni L., Drusbosky L.M., Bilen M.A. (2021). Detection of Microsatellite Instability via Circulating Tumor DNA and Response to Immunotherapy in Metastatic Castration-Resistant Prostate Cancer: A Case Series. Case Rep. Oncol..

[24] Reimers M.A., Yip S.M., Zhang L., Cieslik M., Dhawan M., Montgomery B., Wyatt A.W., Chi K.N., Small E.J., Chinnaiyan A.M. (2020). Clinical Outcomes in Cyclin-dependent Kinase 12 Mutant Advanced Prostate Cancer. Eur. Urol..

[25] Shaya J., Nonato T., Cabal A., Randall J.M., Millard F., Stewart T., McKay R.R. (2021). Analysis of the Prognostic Significance of Circulating Tumor DNA in Metastatic Castrate Resistant Prostate Cancer. Clin. Genitourin. Cancer.

[26] Garrido Castillo L.N., Mejean A., Vielh P., Anract J., Decina A., Nalpas B., Benali-Furet N., Desitter I., Paterlini-Bréchot P. (2022). Predictive Value of Circulating Tumor Cells Detected by ISET^®^ in Patients with Non-Metastatic Prostate Cancer Undergoing Radical Prostatectomy. Life.

[27] Labidi S., Jiao B., Tam S., Fallah P., Salehi A., Rajan R., Alameldin M., Brimo F., Foulkes W.D., Papadakis A.I. (2025). Evaluating the Role of Liquid Biopsy to Detect Pathogenic Homologous Recombination Repair (HRR) Gene Alterations in Metastatic Prostate Cancer. Cancers.

[28] Dong Q., Chen C., Hu Y., Zhang W., Yang X., Qi Y., Zhu C., Chen X., Shen X., Ji W. (2023). Clinical application of molecular residual disease detection by circulation tumor DNA in solid cancers and a comparison of technologies: Review article. Cancer Biol. Ther..

[29] Danesi R., Lo Y.M.D., Oellerich M., Beck J., Galbiati S., Del Re M., Lianidou E., Neumaier M., van Schaik R.H.N. (2021). What do we need to obtain high quality circulating tumor DNA (ctDNA) for routine diagnostic test in oncology? Considerations on pre-analytical aspects by the IFCC workgroup cfDNA. Clin. Chim. Acta.

[30] Baden J., Lin C.-H.J., Anfora A.T., Beer J., Bisselou K., Bungo J., Corner A.S., Danek T., Dickey J., Godsey J.H. (2026). Generic protocols for analytical validation of tumor-informed circulating tumor DNA assays for molecular residual disease: The Blood Profiling Atlas in Cancer’s Molecular Residual Disease Analytical Validation Working Group consensus recommendation. JCO Precis. Oncol..

[31] European Parliament and Council of the European Union (2017). Regulation (EU) 2017/746 of 5 April 2017 on in vitro diagnostic medical devices and repealing Directive 98/79/EC and Commission Decision 2010/227/EU. Off. J. Eur. Union.

[32] Charrière K., Pazart L. (2023). Clinical evidence requirements according to the IVDR 2017/746: Practical tools and references for underpinning clinical evidence of IVD-MDs. Clin. Chem. Lab. Med..

