# Supplementary material for: Liquid Biopsy in Non-Metastatic Prostate Cancer: Clinical Evidence and Future Directions
Source: Cancers (Basel). 2026 Feb 28;18(5):800. doi: 10.3390/cancers18050800 (PMC12984391; doi:10.3390/cancers18050800)
Supplement: Supplementary file 1 [file cancers-18-00800-s001.zip › cancers-4147202-supplementary.pdf]

Study (1st author, year)	Biomarker / platform	Assay class	Analytical LoD (reported/derivable)	Sens/spec (if available)	UMI usage	Endpoint(s)
**Pal, 2015**	**CTCs** by **CellSearch** (EpCAM-enrichment; modified pre-analytic processing; fragments also counted)	Tumor-naïve	**≥1 CTC / 7.5 mL** (enumeration threshold; platform-based)	Not reported	No	**BCR** (biochemical recurrence)
**Khurana, 2013**	**CTCs** by **CellSearch**	Tumor-naïve	**≥1 CTC / 7.5 mL** (explicitly used as "lowest LoD")	Not reported	No	None (feasibility / prevalence study; no OS/MFS/BCR modeling)
**Grisanti, 2016**	**CTCs** by **CellSearch** at PSA recurrence	Tumor-naïve	**≥1 CTC / 7.5 mL**	Not reported	No	None (CTC prevalence at biochemical recurrence; no OS/MFS)
**Hennigan, 2019**	ctDNA by **ULP-WGS** (SCNA) + **patient-specific targeted resequencing**	Tumor-informed (for targeted resequencing); tumor-naïve (ULP-WGS SCNA screen)	Benchmarking shows **robust detection at ~10 mutant copies** in spike-ins; localized PCa samples largely below detection	Not reported	**Yes** (7-bp molecular identifiers in custom library)	**BCR** (follow-up reported; ctDNA largely not detectable pre-RP)
**Lau, 2020**	ctDNA by **personalized capture panel** (tumor WGS-informed) + separate **TP53 TAm-Seq** screening cohort	Tumor-informed (personalized panel); tumor-naïve (TP53 TAm-Seq)	Not stated as a single LoD; sequencing depth ~**40,000× raw** for personalized panel; designed for very low VAF detection	Not reported	**Yes** (TruSight Oncology **UMI** reagents for personalized panel); TP53 TAm-Seq: not UMI-based	**MFS** (metastasis-free survival) *(primary reported)*
**Pope, 2024**	ctDNA by **INVAR** (integration of variant reads; patient-specific mutation set; SureSelect XT HS)	Tumor-informed	Empirical ctDNA fractions reported down to **IMAF ~1.17×10⁻⁵** (range includes 10⁻⁵–10⁻³)	**95% specificity** threshold explicitly used for ctDNA-positivity classification	Likely **Yes** (SureSelect XT **HS** chemistry is UMI-based; method is explicitly error-suppressed)	**BCR**, **MFS**
**Fei, 2023**	ctDNA by **targeted multigene NGS panels** (two panel versions over time)	Tumor-naïve	Defined pragmatically as **ctDNA fraction >0%**; technical LoD not stated	Not reported	Not specified	**BCR** (reported as bPFS / recurrence after RP)
**Kluge, 2024**	ctDNA by **low-pass WGS** CNV-based quantification (radiogenomic comparison vs PSMA PET)	Tumor-naïve	Not stated as LoD; discovery defined by CNV calling/statistical significance filters	Not reported	**Yes** (3-bp random sequence used as UMI in adapters)	**OS**
**Zang, (abstract)**	ctDNA by **tumor-guided multiplex PCR + hybrid-capture normalization + UMIs** (MRD-focused)	Tumor-informed	Detectable tumor fraction reported as low as **~0.004–0.006%** (post-op timepoints)	Not reported	**Yes**	**BCR** (exploratory association with PSA relapse)
